# Special Issue: New Advances in Bioinformatics and Biomedical Engineering Using Machine Learning Techniques, IWBBIO-2022

**DOI:** 10.3390/genes14081574

**Published:** 2023-08-01

**Authors:** Olga Valenzuela, Francisco Ortuño, Alfredo Benso, Jean-Marc Schwartz, Alexandre G. de Brevern, Ignacio Rojas

**Affiliations:** 1 Department of Applied Mathematics, University of Granada, 18071 Granada, Spain; olgavc@ugr.es; 2 Department of Computer Architecture and Computer Technology, Information and Communications Technology Centre (CITIC-UGR), University of Granada, 18010 Granada, Spain; fortuno@ugr.es; 3 Systems Biology Group, Dip. Automatica e Informatica, Politecnico di Torino, Corso Duca degli Abruzzi, 24, 10129 Torino, Italy; 4 School of Biological Sciences, Faculty of Biology, Medicine and Health, University of Manchester, Oxford Road, Manchester M13 9PT, UK; 5 Université de Paris, INSERM, BIGR, F-75014 Paris, France

## 1. Introduction

Bioinformatics is revolutionizing Biomedicine in the way we treat and diagnose pathologies related to biological manifestations resulting from variations or mutations of our DNA. This is why, step by step, a transition is being made from a more observational and diagnostic medicine to a Personalized and Precision Medicine (PPM). The aim is to treat each patient in a personalized way, based on the diagnosis on their own genetic profile, in order to attack the pathology more efficiently. Among all diseases, cancer is one of the most benefit ones from PPM, since in its basis it is a genetic disease. The ever closer possibility of selecting individualized treatments for each patient based on the presence of certain biomarkers opens up enormous expectations in improving the prognosis of these patients. The deep genome and transcriptome sequencing facilitate the discovery of new more reliable molecules for the diagnosis and evolution of the disease.

PPM therefore requires new biological findings, the application of innovative technologies, the comprehensive review of key biomarkers, including new ones, and the application of integrative analytical methodologies and advanced computational platforms. Thus, all the expected revolution of PPM comes with its convergence with artificial intelligence and machine learning techniques. PPM can make use of the characterization of phenotypes and genotypes of patients (for example, molecular profiles, information on their gene expression, medical images, medical signals of biosensors, data on lifestyle, clinical history, etc.) to design the optimal therapeutic strategy at a given time, and/or determine the predisposition towards the disease and/or develop adequate prevention at the right time, all with the aid of massive data analysis and processing, through the use of intelligent techniques.

The advance of artificial intelligence and its impact on our society is currently being a great revolution. The use of machine learning systems and artificial intelligence elements is present in our daily lives more than we are aware of. These tools are also increasingly being used in the field of health. The evolution of bioinformatics, with increasingly accurate, adaptive and intelligent tools, which are able to work with a large number of data (even heterogeneous data sources), is becoming a reality in cutting-edge research in the field of bioengineering and bioinformatics.

This SI highlights current bioinformatics and biomedical approaches, in which in more of the case, the use of advanced tools, in which machine learning occupies a position of relevance, together with advanced computing platforms, allow further progress in this exciting field of research. The main insights from the contributions to this SI are summarised below.

## 2. Contributions

In the paper entitled “Papillary Thyroid Carcinoma: A thorough Bioinformatic Analysis of Gene Expression and Clinical Data”, by Iván Petrini et al. [1], the main goal was to present a comprehensive analysis of Papillary Thyroid Carcinoma (PTC), which is the is the most common type of cancer affecting the thyroid. The contribution include stages for feature selection, hypothesis testing, and classification using machine learning techniques. Two datasets from Gene Expression Omnibus (GEO) and The Cancer Genome Atlas (TCGA) dataset were studied. A final small cluster of genes of interest were obtanined: PTGFR, ZMAT3, GABRB2, and DPP6.

Akram Vasighizaker et al. [2], in the contribution entitled: “Cell Type Annotation Model Selection: General-Purpose vs. Pattern-Aware Feature Gene Selection in Single-Cell RNA-Seq Data” presents a comparative analysis of the use of different machine learning methods in single-cell RNA sequencing (scRNA-seq) scRNA-seq. Recent studies on scRNA-seq technology have been widely applied in biological research and drug discovery. Before investigating the functionality of individual cells in depth for pathological purposes, identification of cell types is an essential step. Recently, several machine learning methods have been developed to identify cell types. Due to the lack of sufficient annotated datasets, supervised techniques have not been commonly used in scRNA-seq studies. Classification methods use feature selection algorithms to improve the accuracy of cell type prediction by searching for marker genes among many implicated genes in high-dimensional datasets. The results of the experiments on three standard scRNA-seq datasets indicate that XGBoost automatically annotates cell types in a simpler and more scalable framework.

As presented by Dobrovolny et al. [3] entitled: “Study on Sperm-Cell Detection Using YOLOv5 Architecture with Labaled Dataset”, infertility has become a serious health problem in recent years. Sperm morphology, sperm motility and sperm density are the most critical factors for male infertility. Therefore, sperm motility, density, and morphology are examined in semen analysis, which is performed by laboratory professionals. It is easy to make a mistake when using subjective analyses based on laboratory observations. To reduce the influence of specialists on semen analysis, this article proposes a computerized approach to estimating sperm count. The number of active sperm in semen is determined by object recognition methods in this study, which focuses on sperm motility. The proposed strategy was tested using data from the Visem dataset provided by the Association for Computing Machinery. The authors created a small custom sample dataset to show that our network will be able to detect sperm in images. The best result without Supertune is Mean Average Precision of 72.15.

In the paper “An Iterative Unsupervised Method for Gene Expression Differentiation” by Olga Georgieva [4] is focoused in the challenge of selecting differentially expressed genes. Different methods for RNA-seq data analysis that identify different genes according to their expression levels have been proposed basically on statistical data analysis. There is no agreement among the applied methods as different results are produced by the distinct methods. In this contribution, a comparative study of the clustering methods used for gene expression analysis is introduced to explain the choice of the clustering algorithm implemented in the method. An investigation of different distance measures is presented to reveal those that increase the efficiency of the method in finding the real data structure.

In the contribution “Omics Data Preprocessing for Machine Learning: A Case Study in Childhood Obesity”, by Álvaro Torres-Martos et al. [5], the main goal is the analysis of the childhood obesity, which is a multifactorial disease influencing the development of a range of metabolic disorders, where adipose tissue has been proved to be fundamental. The authors present machine learning predictive models with multi-omics human data, and provides a collection of best practices and guidelines that could be applied to other human diseases with complex fundamentals (e.g., obesity).

Richard Oliver Matzko et al. [6], in the paper: “Novel Ground-Up 3D Multicellular Simulators for Synthetic Biology CAD Integrating Stochastic Gillespie Simulations Benchmarked with Topologically Variable SBML Models” the authors are focoused in the field of Synthetic Biology. Existing synthetic biology CAD approaches are perceived to be limited in terms of population-level behaviour, and this work explored the in silico challenges raised from biological and computational perspectives.

The Single-cell Transposase-Accessible chromatin assay by sequencing (scATAC-seq) is fast becoming a powerful technology for assessing the epigenetic spectrum landscape of thousands of cells. In the paper “GAGAM v1.2: An Improvement on Peak Labeling and Genomic Annotated Gene Activity Matrix Construction” by Lorenzo Martini et al. [7] presents a novel methodology to improve the Gene Activity Matrix (GAM) approach, which links accessibility data to genes, proposing an improved version of the Genome-Annotated Gene Activity Matrix (GAGAM) concept.

There is a growing interest in the scientific community in the generation of synthetic bulk and single cell RNA-seq data. However, there is no comprehensive and consistent approach to evaluate the different characteristics and performance metrics of data generation methods, especially in terms of “closeness” to the original data. The paper: “A Framework for Comparison and Assessment of Synthetic RNA-Seq Data” by Felitsiya Shakola et al. [8] presents a general framework for evaluating synthetically generated RNA-seq data, comparing the properties of these data, and selecting a data generation tool suitable for a specific set of study objectives.

Finally, in the paper by Gaetan Senelle et al. [9], with title: “Investigating the Diversity of Tuberculosis Spoligotypes with Dimensionality Reduction and Graph Theory”, analyze the spoligotype. The spoligotype is a graphical description of the CRISPR locus present in Mycobacterium tuberculosis, which has the peculiarity of having only 68 possible spacers. This paper exploits in depth the description of the variety of spoligotypes by means of a graph, and shows to what extent such a description can be meaningful.
